# Effects of Zumba^®^ and Aquagym on Bone Mass in Inactive Middle-Aged Women

**DOI:** 10.3390/medicina55010023

**Published:** 2019-01-21

**Authors:** Esther Ubago-Guisado, Javier Sánchez-Sánchez, Sara Vila-Maldonado, Leonor Gallardo

**Affiliations:** 1 University of Castilla-La Mancha, IGOID Research Group, 45071 Toledo, Spain; javier.sanchez2@universidadeuropea.es (J.S.-S.); leonor.gallardo@uclm.es (L.G.); 2 European University, School of Sport Sciences, 28670 Madrid, Spain; 3 University of Castilla-La Mancha, GENUD Toledo Research Group, 45071 Toledo, Spain; Sara.vila@uclm.es

**Keywords:** bone mineral content, bone mineral density, DXA, osteoporosis, physical activity

## Abstract

*Background and objectives*: Regular exercise may stimulate bone formation and reduce the loss of bone mass in premenopausal women. This study aims to evaluate the effect of high-impact physical activity (Zumba^®^) and low-impact physical activity (Aquagym) on bone mass in inactive middle-aged women. *Materials and methods*: Fifty-five healthy inactive women (30–50 years old) were recruited in Spain in 2016 and were randomly allocated into one of three groups: High impact group (HIG: *n* = 15), low impact group (LIG: *n* = 12) and control group (CG: *n* = 28). HIG and LIG were recruited from Madrid and the CG from Toledo. HIG and LIG completed a 12-week intervention program with three 40′ sessions per week of Zumba^®^ or Aquagym; respectively. Dual energy X-ray absorptiometry (DXA) measured bone mineral content (BMC) and areal bone mineral density (aBMD) at total body less head (TBLH), lumbar spine and right hip. *Results*: Post-intervention adjusted data showed no significant differences in BMC between any of the groups nor in aBMD between HIG and LIG. Interestingly; significant differences for the HIG vs. CG were found in the change in total hip aBMD (1.76% vs. −0.44%), femoral neck aBMD (1.80% vs. −2.71%), and intertrochanter aBMD (2.03% vs. −0.50%). Moreover, significant differences for the LIG vs. CG were also found in the change in femoral neck aBMD (−0.54% vs. −2.71%). *Conclusions*: The regular practice of Zumba^®^ and Aquagym might reduce the progressive deterioration of bone mass in inactive middle-aged women

## 1. Introduction

Women not only have a high risk of developing osteoporosis, but also of suffering bone fractures during menopause [1]. In 2010, more than 22 million European women over the age of 50 years had osteoporosis [2]. The hip and the lumbar spine are the regions with the greatest incidence of fracture [3]. Premenopausal women lose between 0.25 and 1% of areal bone mineral density (aBMD) each year [4]. Thus, preventative strategies (i.e., healthy lifestyles) that contribute to reducing the risk of suffering osteoporosis and potential bone fractures are needed [1]. The scientific evidence shows that physical activity may prevent the loss of bone mass during menopause [5], but more investigations are necessary to find out the effect of different types of exercise for reducing the risk of osteoporosis [6].

Through physical activity, bones undergo a remodelling process to adapt to the mechanical load that the bone supports [7]. As a consequence the bone mineral content (BMC) and aBMD increase, along with bone trabecular adaptations [8]. Regular physical activity during menopause seems to improve bone metabolism and bone health, delaying the onset of osteoporosis [6]. Therefore, regular exercise may stimulate bone formation and reduce the loss of bone mass in premenopausal women [3].

A meta-analysis in postmenopausal women concluded that exercise was very effective to improve lumbar spine and femoral neck aBMD [5]. These benefits in bone mass were also observed in a meta-analysis of premenopausal women by Kelley, et al. [9]. In addition, a meta-analysis by Zhao, Zhao and Zhang [3] concluded that the skeletal response of the lower limbs to plyometric exercises was positive in premenopausal women. In addition, the aBMD of the femoral neck may be positively stimulated [8]. Likewise, the residual benefits of a high impact exercise intervention have been shown to be maintained even after 3.5 years [10].

Nowadays, Zumba^®^ is considered one of the most popular high-impact physical activities among women [11]. However, few studies have investigated its benefits on bone mass [12]. It seems reasonable hypothesizing that Zumba^®^ might stimulate the bones, due to the force and stress produced during its practice [13]. Previous studies affirm that the osteogenic stimulus depends on the presence of mechanical stress on the bone [14,15], and therefore the practice of Zumba^®^, which involves intense and high-impact actions, could be beneficial [11].To date, the few studies using Zumba^®^ as an intervention program to improve bone mass have not reached statistical significance [11,16] after 12 and 40-weeks of intervention. Nonetheless, the authors indicated that Zumba^®^ had the potential for improving bone mass, and also other health markers, such as cardiorespiratory fitness and the accumulation of fat mass. In this line, a significant reduction in the waist and hip circumferences has been found following 12 and 16 weeks of Zumba^®^ practice [17,18] and also an increment in lean mass [17].

Aquagym, as a form of physical activity in water, is also very popular among middle-aged women [6,19]. However, it may not be considered as an osteogenic exercise, due to the low mechanical load that the bone receives during its practice [20]. So far, most investigations in aquatic environments have been conducted in swimmers. Some studies have proven that swimmers have less bone mass than those who practice high impact sports during youth [21,22] and adulthood [23]. Alternatively, other studies have found no differences in bone mass between swimmers and control groups in growing populations [24]. To the best of our knowledge, the effect of Aquagym on bone mass has yet to be investigated.

Therefore, the aim of this study was to evaluate the effect of twelve weeks of high-impact physical activity (Zumba^®^) and low-impact physical activity (Aquagym) on bone mass in inactive middle-aged women after controlling for key confounders.

## 2. Materials and Methods

### 2.1. Participants

The sample was made up of 55 inactive Spanish women between the ages of 30–50 years (mean and standard deviation, 43.1 ± 5.9 years old). The inclusion criteria were: Not having reached menopause, not suffering or have suffered any illness that affect bone health, not taking any supplement that may affect calcium acquisition or absorption, and not accumulating more than 150 min of moderate physical activity per week or more than 75 min of vigorous physical activity per week [25]. The sample size was calculated using G-Power 3.1 software, for ANOVA, repeated measures, within-between interaction. Based on a 90% power, alpha level set at 5%, assuming a correlation among repeated measures of 0.7, 3 groups and 2 repetitions, 33 participants (11 per group) were needed.

Leaflets were used to recruit participants. Leaflets were left at information desks and boards within the University´s Faculties and Hospital. All participants gave their written consent to participate in the study and were informed verbally and in a written manner of the experimental procedures and the risks associated. The study was approved by the ethical committee of the CEIC of the Madrid Community (P2016/UEM33) and carried out in accordance with the Helsinki Declaration. All evaluations were performed from March to June 2016.

### 2.2. Experimental Design and Training Intervention

Participants were randomly assigned into one of the two exercise groups: High impact group (HIG: *n* = 15) and low impact group (LIG: *n* = 12). In addition, a control group (CG: *n* = 28) was recruited in parallel. The study was designed as a controlled trial and baseline and post-measures were taken. All participants completed the intervention and no drop outs were registered.

Both training programs (high impact and low impact) lasted 12 weeks. HIG completed 3 × 40′ sessions per week of active dancing (Zumba^®^), considered as a moderate/vigorous intensity exercise that includes jumps and changes of direction. On the other hand, the LIG completed 3 × 40′ sessions per week of moderate/vigorous intensity Aquagym, including jumps and changes of direction. Experienced trainers supervised all training sessions and provided with technical supervision, controlled intensity, duration of the activity and ensured a safe training environment. A one-week familiarization period was given to all participants. A maximum of 5 missing sessions (out of 36) was allowed for women to be considered valid participants for the study. In this regard, all women met this criterion and the compliance rate was >85%.

### 2.3. Anthropometry and Dual Energy X-ray Absorptiometry

Weight (kg) and height (cm) were measured using the SECA scale (model 711; SECA GmbH & Co, KG, Hamburg, Germany). Body mass index (BMI) was calculated using the formula: BMI (kg·m^−2^) = body weight (kg)/body height (m)^2^.

A DXA scanner (Hologic Series Discivery QDR, Software Physician’s Viewer, APEX System Software Version 3.1.2. Bedford, MA, USA) was used at baseline and post-intervention to measure BMC (g) and aBMD (g/cm^2^). Three scans were performed to obtain data for the whole body (including legs, arms and total body less head), lumbar spine (L1–L4) and right hip (including trochanter, intertrochanter, femoral neck, and total hip). The DXA equipment (Hologic Series Discovery QDR, Software Physician’s Viewer, APEX System Software Version 3.1.2. Bedford, MA, USA) was calibrated prior to each testing day by using a lumbar spine phantom following manufacturer recommendations. The positioning of the participants and the analyses of the results were undertaken according to the International Society for Clinical Densitometry [26]. Precision studies in people aged 42–70 year old have shown the coefficient of variation for DXA to be 1.71% for the aBMD of the lumbar spine, and 1.05% for the total hip aBMD [27].

### 2.4. Statistical Analysis

Data were analyzed using the statistical analysis software SPSS V19.0 for Windows (SPSS Inc, Chicago, IL, USA) and the statistical significance level was set at *p* < 0.05. The normality of the data was checked with standard tests. Descriptive data are presented as mean and standard deviation. One-way analysis of variance (ANOVA) with Bonferroni post-hoc was performed to detect between-group differences in the descriptive characteristics at baseline. In addition, repeated measures analysis of variance (ANOVA) was used to test within-group changes from baseline to post-intervention. Finally, a one-way analysis of covariance (ANCOVA) with Bonferroni post-hoc was used to examine between-group differences in changes of bone variables using age, stature, lean mass and bone outcomes at baseline as confounders [28]. The adjusted percentage of difference with 95% confidence intervals (95% CI) are shown to quantify the magnitude of the differences. In addition, effect size (ES; Cohen’s d) were calculated and evaluated following the criteria: 0–0.2 = trivial, 0.2–0.5 = small, 0.5–0.8 = moderate and 0.8 = large [29].

## 3. Results

Descriptive characteristics of the participants at baseline are presented in Table 1. Women in the HIG had significantly lower BMI than those in the CG. Women in the CG had significantly higher values in total hip BMC compared to those in the HIG. Also, women in the LIG had significantly higher total body less head (TBLH) aBMD compared to those in the CG.

Table 2 shows within-group raw bone values at baseline and post intervention. After the 12-week program, the HIG significantly improved the legs BMC (ES = 0.14), and intertrochanter aBMD (ES = 0.12). The CG significantly lost TBLH BMC and aBMD (ES = 0.14 and 0.40, respectively), LS BMC (ES = 0.32), and FN aBMD (ES = 0.13). However, no significant changes in BMC or aBMD were observed in the LIG.

Table 3 shows between-group bone-adjusted differences after the intervention using age, stature, lean mass and the baseline bone outcomes as confounders. No significant differences in BMC between any of the groups nor in aBMD between HIG and LIG were found. Interestingly, significant differences for the HIG vs. CG were found in the change in total hip aBMD (1.76% vs. −0.44%), femoral neck aBMD (1.80% vs. −2.71%), and intertrochanter aBMD (2.03% vs. −0.50%). Moreover, significant differences for the LIG vs. CG were also found in the change in femoral neck aBMD (−0.54% vs. −2.71%).

## 4. Discussion

The present study is the first to analyze the osteogenic effects of Zumba^®^ (HIG) and Aquagym (LIG) in inactive middle-aged women. The main findings after 12 weeks of training were that 1) regular practice of Zumba^®^ and Aquagym maintained BMC and aBMD in most regions and even improved it at some sites with the practice of Zumba^®^, and 2) post-intervention changes showed greater adjusted aBMD in the hip regions for the HIG and LIG compared to those in the CG. Interestingly, women in the inactive group lost BMC and aBMD at some sites over 12 weeks. Previous investigations have proven the importance of exercise on the bones showing that physically active participants had significantly higher aBMD than their sedentary peers of the same age [30,31,32]. Regular exercise positively affects bone metabolism and significantly improves bone health in premenopausal women [9]. This favorable effect is caused by the physical stress and weight loading that promotes bone modelling and remodeling to maintain bone mineralization [31,33].

After 12 weeks of intervention, raw data showed that women who practiced high impact exercise (Zumba^®^) increased their legs BMC and their intertrochanter aBMD. This may be due to the fact that exercises in the forms of jumps are known to improve bone health and reduce bone loss [3,34]. In addition, jumps are associated with ground reaction forces four to seven times body weight [35]. This links very well with the Mechanostat theory, which postulates that the bone adapts to the force and stress produced during exercise, and therefore increases its strength [13]. This mechanism regulates the modelling and remodeling processes of the bone, being affected by the mechanical forces applied to the skeleton (force, pressure and torsion) [36]. In this regard, the increase in maximum muscle strength during growth or the response to the increased load will affect the mass, size and strength of the bone through an increase in lean mass [37,38].

Women engaged in Zumba^®^ (HIG) and Aquagym (LIG) improved or maintained their BMC and aBMD at different sites. Our adjusted data agrees with the study by Zhao, Zhao and Zhang [3], who concluded that physical activity was efficient in the increase and/or maintenance of aBMD in premenopausal women. Also, a meta-analysis carried out in the same population showed that exercise benefits lumbar spine and femoral neck aBMD in premenopausal women [9]. When we compared the post-intervention changes, we observed that women engaged in Zumba^®^ increased their aBMD at the intertrochanter, femoral neck and total hip by 1.8% to 2.0% and these changes were significantly different from those observed in the inactive women who lost had their aBMD reduced between −0.4% to −2.7%. In addition, a slight reduction (−0.5%) in femoral neck aBMD was observed in women who practiced Aquagym, while those who were inactive being significantly different to the reduction observed in the group of inactive women (−2.7%). Various lines of evidence have shown positive changes in the aBMD after performing plyometric exercise interventions [34,39,40,41], especially at the femoral neck [42,43], which is a key region in the diagnosis of osteoporosis. In addition, according to the study by Kontulainen, Heinonen, Kannus, Pasanen, Sievanen and Vuori [10], this advantage could be maintained up to three years after stopping the practice of exercise. Therefore, our data support these two type of exercises as effective strategies to stimulate bone formation and reduce the rate of bone mass lost in premenopausal women [3].

On the other hand, women in the LIG (Aquagym) maintained their BMC and aBMD at all sites, except at the femoral neck where a very small reduction in aBMD of −0.5% was observed. To the best of our knowledge, this is the first study investigating the effect of Aquagym on bone mass and most investigations in aquatic environments have been conducted in swimmers. This type of exercise shares common characteristics with swimming, due to its hypogravity and lack of impact. In a review focused on bone health in swimmers, the authors observed that swimming did not seem to negatively affect bone mass [21], which is similar to what we found with Aquagym. Taking into account that the remodeling process in middle-aged women is characterized by a continuous deterioration of bone mass year after year [4], the fact that women who practiced Aquagym did not significantly lose bone mass suggests that this type of exercise may help to slow down the deterioration of bone mass with age. In this regard, the muscle-bone interaction during exercise, even without impact, might cause an osteogenic effect on the bone or maintain it [44], due to the action of the muscle stress that stimulate bone tissue [45]. Even so, some reviews of the literature have demonstrated that high impact sports, such as football, basketball, handball, squash, running, tennis, ice-hockey, badminton, volleyball and weight-lifting seem to be more osteogenic than non-impact sports like swimming or cycling, in children [22,46], young adults [20] or older adults [47].

This study has some limitations that have to be mentioned. Biochemical blood markers were not measured, which would have provided with additional information on bone changes. Also, no data were collected about dietary habits (e.g., calcium or vitamin D intake), which might have influenced the results. Physical activity (i.e., using accelerometry) was not recorded and this could have affected our findings; however, the participants in this study were inactive. DXA is a gold-standard device used in the diagnosis of osteoporosis, however, using computed tomography techniques would have been ideal to observe small cortical and trabecular changes. The duration of the intervention was short (12 weeks), but enough to observe some adaptations. Since Zumba^®^ and Aquagym have become very popular studies among women, future studies with a longer intervention period and follow-up measures are needed to better understand bone changes in this population and the residual effects.

## 5. Conclusions

The regular practice of Aquagym, and more importantly the practice of Zumba^®^ seems to reduce the progressive deterioration of bone health in inactive middle-aged women. These findings highlight the importance of implementing exercise programs in pre-menopausal women to slow down the loss of bone mass with ageing.

## Figures and Tables

**Table 1:** Descriptive characteristics of the participants at baseline.

	HIG ^a^	LIG ^b^	CG ^c^
N	15	12	28
Age (year)	41.3 ± 5.1	42.2 ± 7.6	45.8 ± 5.1
Stature (cm)	164.4 ± 4.0	162.2 ± 6.8	159.9 ± 6.1
Body mass (cm)	60.5 ± 7.6	67.7 ± 14.1	66.1 ± 13.4
BMI (kg/m^2^)	**22.3 ^c^ ± 2.2**	25.6 ± 4.2	25.9 ± 5.2
Fat mass (kg)	20.72 ± 5.14	26.38 ± 8.46	23.99 ± 8.71
Fat mass (%)	37.54 ± 5.01	42.19 ± 5.60	37.78 ± 7.27
Lean mass (kg)	37.16 ± 3.26	38.35 ± 5.72	39.30 ± 5.68
BMC (g)			
Lumbar spine	58.77 ± 7.47	57.25 ± 11.36	52.89 ± 9.90
Hip total	29.63 ± 4.15	33.42 ± 5.89	**38.23 ^a^ ± 6.94**
TBLH	2094.37 ± 207.33	2239.02 ± 335.65	2030.98 ± 371.75
aBMD (g/cm^2^)			
Lumbar spine	0.989 ± 0.109	1.008 ± 0.140	0.921 ± 0.118
Hip total	0.869 ± 0.113	0.950 ± 0.080	0.942 ± 0.126
TBLH	1.137 ± 0.092	**1.194 ^c^ ± 0.095**	1.075 ± 0.112

Values presented as mean ± SD o percentages; HIG, high impact group; LIG, low impact group; CG, control group; BMI, body mass index; BMC, bone mineral content; aBMD, areal bone mineral density; TBLH, total body less head; Bold numbers and superscript letters denote a significant difference (*p* < 0.05) compared to: ^a^ (HIG), ^b^ (LIG) and ^c^ (CG).

**Table 2:** Unadjusted bone mineral content (BMC) and areal bone mineral density (aBMD) at baseline and post-intervention.

	HIG		LIG		CG	
	Baseline	Post-Intervention	Baseline	Post-Intervention	Baseline	Post-Intervention
BMC (g)						
Lumbar spine	58.77 ± 7.47	58.30 ± 7.57	57.25 ± 11.36	57.54 ± 10.74	52.89 ± 9.90 *	49.87 ± 9.09 *
Trochanter	6.45 ± 1.02	6.46 ± 0.94	7.09 ± 1.22	7.11 ± 1.31	6.76 ± 1.39	6.77 ± 1.38
Intertrochanter	18.88 ± 2.64	19.41 ± 3.36	21.70 ± 4.58	21.65 ± 5.01	27.96 ± 5.47	27.93 ± 5.22
Femoral neck	4.30 ± 0.84	4.35 ± 0.92	4.66 ± 0.76	4.69±0.79	3.48 ± 0.61	3.39 ± 0.68
Hip total	29.63 ± 4.15	30.10 ± 5.04	33.42 ± 5.89	33.48 ± 6.40	38.23 ± 6.94	38.03 ± 7.03
Legs	369.77 ± 47.81	376.53 ± 47.24 *	396.03 ± 61.05	399.63 ± 61.05	345.69 ± 66.87	340.53 ± 62.96
Arms	132.04 ± 14.04	132.79 ± 15.82	148.78 ± 36.71	149.76 ± 36.80	142.67 ± 27.80	142.44 ± 26.41
TBLH	2094.37 ± 207.33	2100.89 ± 201.31	2239.02 ± 335.65	2245.15 ± 332.34	2030.98 ± 371.75 *	1982.92 ± 331.73 *
aBMD (g/cm^2^)						
Lumbar spine	0.989 ± 0.109	0.984 ± 0.112	1.008 ± 0.140	1.018 ± 0.137	0.921 ± 0.118 *	0.884 ± 0.119
Trochanter	0.649 ± 0.100	0.652 ± 0.099	0.690 ± 0.054	0.693 ± 0.052	0.652 ± 0.092	0.656 ± 0.094
Intertrochanter	1.023 ± 0.136	1.040 ± 0.142 *	1.122 ± 0.108	1.127 ± 0.107	1.094 ± 0.141	1.092 ± 0.148
Femoral neck	0.761 ± 0.082	0.771 ± 0.090	0.842 ± 0.093	0.841 ± 0.094	0.745 ± 0.118 *	0.730 ± 0.112
Hip total	0.869 ± 0.113	0.886 ± 0.132	0.950 ± 0.080	0.954 ± 0.085	0.942 ± 0.126	0.940 ± 0.125
Legs	1.171 ± 0.112	1.177 ± 0.095	1.226 ± 0.099	1.231 ± 0.094	1.089 ± 0.117	1.051 ± 0.126
Arms	0.715 ± 0.040	0.710 ± 0.037	0.757 ± 0.106	0.755 ± 0.102	0.744 ± 0.105	0.729 ± 0.088
TBLH	1.137 ± 0.092	1.139 ± 0.083	1.194 ± 0.095	1.192 ± 0.092	1.075 ± 0.112 *	1.030 ± 0.115

Unadjusted values presented as mean ± SD; HIG, high impact group; LIG, low impact group; CG, control group; BMC, bone mineral content; aBMD, areal bone mineral density; TBLH, total body less head.; Statistically significant (* *p* < 0.05) within-group change (repeated measures ANOVA) compared with the baseline.

**Table 3:** Adjusted changes in bone mineral content (BMC) and areal bone mineral density (aBMD).

	HIG ^a^		LIG ^b^		CG ^c^	
	Baseline	Post-Intervention	Baseline	Post-Intervention	Baseline	Post-Intervention
BMC (g)		**% Diff (95%CI)**		**% Diff (95%CI)**		**% Diff (95%CI)**
Lumbar spine	60.65 ± 2.02	−0.04 (−2.47 to 2.38)	57.68 ± 2.04	0.90 (−1.43 to 3.23)	51.70 ± 1.44	−1.52 (−3.26 to 0.21)
Trochanter	6.79 ± 0.28	1.98 (−0.80 to 4.75)	7.13 ± 0.28	1.13 (−1.72 to 3.99)	6.67 ± 0.07	−1.36 (−3.37 to 0.64)
Intertrochanter	19.69 ± 1.06	1.62 (−1.99 to 5.24)	21.71 ± 1.07	−0.56 (−3.86 to 2.73)	27.52 ± 0.76	−0.30 (−2.94 to 2.34)
Femoral neck	4.34 ± 0.20	1.45 (−2.15 to 5.05)	4.66 ± 0.20	0.87 (−3.04 to 4.79)	3.46 ± 0.14	−2.53 (−5.31 to 0.25)
Hip total	30.80 ± 1.34	2.03 (−0.96 to 5.01)	33.46 ± 1.34	0.07 (−2.74 to 2.87)	37.59 ± 0.96	−0.92 (−3.04 to 1.20)
Legs	377.58 ± 12.52	1.35 (−0.67 to 3.36)	397.69 ± 12.66	1.54 (−0.62 to 3.70)	340.80 ± 8.96	−0.34 (−1.85 to 1.18)
Arms	138.13 ± 6.26	0.04 (−1.85 to 1.93)	151.10 ± 6.33	0.85 (−1.10 to 2.80)	138.41 ± 4.48	−0.06 (−1.29 to 1.42)
TBLH	2145.98 ± 70.91	−0.07 (−1.84 to 1.71)	2248.12 ± 71.69	0.72 (−1.15 to 2.58)	1999.43 ± 50.75	−1.62 (−2.93 to −0.32)
aBMD (g/cm^2^)						
Lumbar spine	0.994 ± 0.033	−0.51 (−2.73 to 1.70)	1.007 ± 0.034	1.29 (−0.97 to 3.55)	0.919 ± 0.024	−1.64 (−3.25 to −0.03)
Trochanter	0.683 ± 0.021	1.04 (−0.20 to 2.27)	0.694 ± 0.021	0.80 (−0.47 to 2.06)	0.660 ± 0.003	0.02 (−0.88 to 0.92)
Intertrochanter	1.058 ± 0.036	**2.03** **^c^** **(0.89 to 3.17)**	1.127 ± 0.036	0.52 (−0.65 to 1.69)	1.073 ± 0.026	−0.50 (−1.31 to 0.32)
Femoral neck	0.781 ± 0.027	**1.80** **^c^** **(−0.07 to 3.66)**	0.843 ± 0.027	**−0.54** **^c^** **(−1.47 to 2.55)**	0.734 ± 0.019	−2.71 (−4.09 to −1.33)
Hip total	0.901 ± 0.030	**1.76** **^c^** **(0.24 to 3.29)**	0.954 ± 0.030	0.43 (−1.12 to 1.98)	0.923 ± 0.021	−0.44 (−1.52 to 0.65)
Legs	1.205 ± 0.028	−0.20 (−2.19 to 1.79)	1.231 ± 0.028	1.08 (−1.02 to 3.18)	1.069 ± 0.020	−0.97 (−2.49 to 0.55)
Arms	0.744 ± 0.024	−1.50 (−3.22 to 0.22)	0.767 ± 0.024	−0.43 (−2.20 to 1.33)	0.724 ± 0.017	−0.42 (−1.66 to 0.82)
TBLH	1.161 ± 0.028	−0.14 (−1.77 to 1.50)	1.199 ± 0.028	−0.49 (−1.25 to 2.24)	1.060 ± 0.020	−1.75 (−2.99 to −0.51)

Adjusted values presented as mean ± SE; For baseline data age, stature and lean mass were used as covariates; For post-intervention data, age, stature, lean mass and bone outcomes at baseline were used as covariates; HIG, high impact group; LIG, low impact group; CG, control group; BMC, bone mineral content; aBMD, areal bone mineral density; TBLH, total body less head; Bold numbers and superscript letters denote a significant difference (*p* < 0.05) between-group change (ANCOVA; Bonferroni adjusted posthoc test) compared to: ^a^ (HIG), ^b^ (LIG) and ^c^ (CG).
